# Tenofovir Disoproxil Fumarate Is Superior to Entecavir in Reducing Hepatitis B Surface Antigen for Chronic Hepatitis B in China: 2-Year Comprehensive Comparative Result of a Matched Comparative Study

**DOI:** 10.3389/fmed.2021.637126

**Published:** 2021-03-15

**Authors:** Sisi Yang, Xueqing Ma, Chengwei Cai, Huanqiu Wang, Fenqiang Xiao, Chengbo Yu

**Affiliations:** ^1^State Key Laboratory for Diagnosis and Treatment of Infectious Diseases, National Clinical Research Center for Infectious Diseases, Collaborative Innovation Center for Diagnosis and Treatment of Infectious Diseases, The First Affiliated Hospital, Zhejiang University School of Medicine, Hangzhou, China; ^2^Department of Neurosurgery, The Second Affiliated Hospital, Zhejiang University School of Medicine, Hangzhou, China; ^3^Department of Emergency Surgery, Tongji Medical College, Union Hospital, Huazhong University of Science and Technology, Wuhan, China

**Keywords:** chronic hepatitis B, tenofovir disoproxil fumarate, entecavir, hepatitis B surface antigen, hepatocellular carcinoma, creatinine

## Abstract

**Aim:** Tenofovir disoproxil fumarate (TDF) and entecavir (ETV) are equally recommended as the first-line antiviral treatments for chronic hepatitis B (CHB) at present. We aimed to compare the long-term efficacy and safety between ETV and TDF therapy in CHB patients who had not received nucleoside analog treatment.

**Method:** In this single-center retrospective study, 414 patients who received ETV (290 patients) or TDF (124 patients) therapy at our center from January 2017 to May 2019 were included. To reduce the imbalance of baseline variables, propensity score matching (PSM) was employed to yield 124 pairs of patients at a ratio of 1:1 based on the treatment regimen.

**Result:** After PSM, the cumulative rate of patients who achieved complete virological response (CVR) was not different by drug therapy at each inspection time (1, 3, 6, 12, 18, and 24 months). Subgroup analysis on HBeAg status and level of HBV DNA demonstrated that evolution of proportion of achieving CVR was not significantly different between groups. Despite the insignificant incidence of HBsAg seroclearance in either group, patients in TDF group achieved higher on-treatment HBsAg decline at each inspection time (1, 3, 6, 9, 12, 18, and 24 months), 0.39, 0.51, 0.61, 0.64, 0.68, 0.76, and 0.91 log IU/mL, respectively; while the corresponding reduction were 0.27, 0.37, 0.40, 0.45, 0.48, 0.55, and 0.66 log IU/mL in ETV group (*p* < 0.05). In subgroup analysis, we found that the significant difference still existed in patients with high baseline HBsAg level (>3 log IU/mL). Additionally, the proportion of patients who achieved on-treatment HBsAg decline >1 log IU/mL in TDF and ETV group was 33.3 and 17.1% (*p* < 0.01) at the 12th month, 44.4 and 29.5% (*p* = 0.03) at the 24th month, respectively. Mean increase in serum creatinine from baseline was 0.10 and 0.08 mg/dL in TDF and ETV group (*p* = 0.11), with no patient experienced acute kidney injury.

**Conclusions:** TDF has higher potency in reducing HBsAg than ETV in this study. Considering the effect still existed in patients with high HBsAg level (>3 log IU/mL), TDF might be a superior therapeutic regimen combining with its relatively safety.

## Introduction

Hepatitis B virus (HBV) has long been a question of great interest in a wide range of fields—~248 million people worldwide are estimated to be chronically infected with HBV and 4.5 million new infections annually (1, 2). Without intervention, patients with chronic hepatitis B (CHB) are at high risk of developing serious complications, such as liver cirrhosis (LC), liver failure, and hepatocellular carcinoma (HCC), causing about 650,000 deaths a year from HBV-related liver failure (3–5). Recent guidelines recommend antiviral treatment with nucleos(t)ide analogs (NAs) and interferon (IFN) for CHB patients (6–8). The biggest obstacle for NAs or IFN to eliminate HBV mainly attributes to the formation and persistence of covalently closed circular DNA (ccc DNA), a replication template with full virus functionality, inside nucleus of hepatocytes. In addition, HBV DNA integration into host genome also affects the efficacy of drugs to some extent, unlike ccc DNA, which replicates as cells divide (9–12). Current antiviral therapeutic goals were aimed at improving liver inflammation, suppressing HBV replication and thus decreasing the incidence of liver complications (13).

In the last two decades, NAs have been widely used for the antiviral treatment of CHB due to potent antiviral effect, high genetic barriers against the formation of drug resistance, and low toxicity (14, 15). Currently approved NAs agents for CHB include nucleoside analogs (lamivudine, telbivudine, ETV) and nucleotide analogs (adefovir dipivoxil, TDF, tenofovir alafenamide) worldwide. These drugs have been proven to be effective in stalling the progression of disease and improving prognosis in clinical practice for many years, differing in antiviral and clinical efficacy, drug resistance, tolerance, and safety, with ETV and TDF recommended as the first-line oral antiviral agents in current clinical practice guidelines (6–8, 15–19).

However, comparison of efficacy between ETV and TDF is still controversial. It is reported in a large proportion of studies including comparative studies and meta-analysis that TDF and ETV are comparable in treatment response, preventing occurrence of hepatocellular carcinoma and safety (20–26). A small number of studies suggest otherwise. A recent Korean nationwide population cohort study enrolling 24156 CHB patients (15) showed that TDF might be more potent in reducing the risk of hepatocellular carcinoma and mortality, while a study conducted in China including 29350 CHB (27) came to the same conclusion. Several meta-analyses were conducted to compare the efficacy between TDF and ETV, finally found that TDF was superior to ETV in lowering HCC risk among patients with CHB, particularly cirrhotic patients (27–29). TamakiSome et al.'s study compared HBsAg reduction between patients switching from long-term entecavir therapy to TDF and patients continuing ETV therapy, indicating TDF was more potent in HBeAg-negative CHB patients than ETV (30). The presence of the conflicting data requires further verification in cohort. In this comparative study, we comprehensively evaluated treatment response, incidence of serious complications and adverse effect in the course of treatment in treatment- naïve CHB patients receiving TDF or ETV as antiviral therapeutic regimen.

## Materials and Methods

### Study Design and Data

We conducted a retrospective cohort study based on medical records, from the First Affiliated Hospital of College of Medicine of Zhejiang University, to compare the efficacy and safety of ETV and TDF in NA-naïve CHB patients. Patients receiving TDF 300 mg or ETV 0.5 mg daily between January 2017 and May 2019 were systematically reviewed with electronic medical records, laboratory inspection, and imaging examination.

The following parameters were recorded in detail: demographic data (age, gender), virological data (HBV DNA level at baseline, 1st, 3rd, 6th, 9th, 12th, 18th, and 24th month), serological data (HBsAg level, HBeAg status and HBeAg level at baseline, 1st, 3rd, 6th, 9th, 12th, 18th, and 24th month), biochemical statistics (alanine transaminase (ALT), baseline and highest serum creatinine level, baseline and minimum blood phosphorus level), and imaging examination (ultrasound, computed tomography, magnetic resonance imaging).

### Inclusion and Exclusion Criteria

Patients who met the following inclusion criteria were enrolled: (1) HBsAg positivity for at least 6 months, (2) oral antiviral therapy naïve, (3) therapy with ETV 0.5 mg/day or TDF 300 mg/day for at least 2 years, (4) regular monitoring every 3 months for the first year of treatment and every 6 months for the second year.

Patients (1) younger than 18 years old, (2) co-infected with hepatitis C, hepatitis D or human immunodeficiency virus, (3) receiving immunosuppressive therapy or with history of immunodeficiency, (4) previously treated with oral antiviral drugs or interferon, (5) pregnant, (6) diagnosed with HCC within the first 6 months of treatment, (7) without regular monitoring records were excluded.

### Serum Assay

HBeAg and serum HBV DNA were measured by enzyme-linked immunosorbent assay (ELISA) (Intec Stone, China) and polymerase chain reaction (PCR)-based Cobas Amplicor HBV Monitor Test (Roche Diagnostics, China) per the manufacturer's instructions, respectively. Serum ALT and creatinine (SCr) were measured by an automatic biochemistry analyzer (Olympus AU5400, Japan) following the standard laboratory procedures. HBV mutations associated with ETV-resistance (rtI169T, rtL180M, rtT184G, rtS202I, rtM204V/I, and rtM250V) were analyzed by PCR pyrosequencing assay if virological breakthrough (VBT) occurred.

### Definitions

We defined complete virological response (CVR) as an undetectable HBV DNA level in serum (HBV DNA ≤ 20 IU/mL or 100 copies/mL by PCR assays), biochemical response (BR) as normalization of high ALT level, HBsAg complete serological response was as HBsAg <20 IU/mL, early virological response (EVR) as suppression of HBV DNA to <420 IU/mL at 3 months, HBsAg partial serological response as at least 1 log IU/mL reduction of HBsAg level, HBeAg seroclearance as HBeAg loss and/or the emergence of anti-HBe anti-body during the antiviral treatment. Virological breakthrough (VBT) was determined as at least 1 log IU/mL rise in serum HBV DNA level above nadir or the reappearance of viremia after achieving CVR, indicating potential drug resistance. Acute kidney injury (AKI) was defined as an absolute increase in SCr from baseline by at least 0.3 mg/dL increase or ≥1.5 times baseline SCr within 48 h without other significant factors leading to renal function impairment, while hypophosphatemia as serum inorganic phosphorus concentration of <0.8 mmol/L (2.5 mg/dl).

The virological, serological, and biochemical responses of the patients treated with TDF and ETV were systematically analyzed. The decreases of HBV DNA and HBsAg over time, and cumulative rate of virological, serological, and biological response during treatment were compared. The primary endpoint was to achieve HBsAg seroclearance.

### Statistical Analysis

Statistical significance was estimated using the Student's *t*-test or Mann–Whitney *U*-test for comparison of continuous variables, while categorical variables were statistically compared using the chi-square or Fisher exact-test. To balance the selected bias, we performed propensity scores matching (PSM) based on logistic regression. After adjusting for drug regimen, the variables used in the model included age, sex, baseline HBV DNA levels, baseline HBsAg level, HBeAg status, baseline HBeAg level, and cirrhosis. We performed nearest-neighbor 1:1 PS matching without replacement, with the caliper width within a range of 0.1 SD. Cumulative probabilities of CVR, BR, serological response, and VBT were estimated using the Kaplan–Meier method with the log-rank test. Univariate and multivariate analyses were performed using Cox proportional hazards regression to determine the independent factors of achieving CVR, HBeAg loss/seroconversion, and HCC development. A *P*-value < 0.05 was considered statistically significant.

## Results

### Characteristics of the Study Patients

Among 414 patients treated with ETV or TDF at the center, between January 2017 and May 2019, who met the inclusion and exclusion criteria, 124 patients were administered with TDF and the remaining 290 patients were administered with ETV. The baseline demographic clinical characteristics of all 414 patients are shown in Table 1, demonstrating baseline demographic and clinical characteristics of the two groups were not comparable at baseline. More patients in TDF group had high baseline HBV DNA, HBeAg, and HBsAg levels, and were HBeAg seropositive; while patients treated with ETV were older, had higher ALT level and more male at the beginning of treatment. To eliminate the influence of the baseline characteristics, we performed the PSM method to match 124 patients in the TDF group with 124 patients in the ETV group by age, sex, baseline HBV DNA levels, baseline HBsAg level, HBeAg status, and cirrhosis. There was no statistical significance detected in post-matched patients (shown in Table 1).

**Table 1:** The baseline characters of pre- and post-matched patients.

	**All patients**			**Matched patients**		
	**ETV (*n* = 290)**	**TDF (*n* = 124)**	***P*-value**	**ETV (*n* = 124)**	**TDF (*n* = 124)**	***P*-value**
Male, *n* (%)	220 (75.9)	72 (58.1)	<0.001	82 (66.1)	72 (58.1)	0.24
Age (years)	38.7 ± 11.1	32.7 ± 8.5	<0.001	34.6 ± 9.4	32.7 ± 8.5	0.09
Serum HBV DNA (log10 IU/mL)	6.74 ± 1.54	7.23 ± 1.22	<0.001	7.22 ± 1.41	7.23 ± 1.22	0.97
HBeAg positivity, n(%)	184 (63.4)	108 (87.1)	<0.001	105 (84.7)	108 (87.1)	0.72
HBeAg (PEIU/ml)	225 ± 183	312 ± 189	<0.001	262 ± 168	312 ± 189	0.05
HBsAg (Log10 IU/mL)	3.70 ± 0.80	4.07 ± 0.80	<0.001	4.05 ± 0.68	4.07 ± 0.80	0.78
LC, *n* (%)	95 (32.8)	18 (14.5)	<0.001	24 (19.4)	18 (14.5)	0.4
Child-Pugh, *n* (%)			0.33			0.83
A	59 (62.1)	9 (50.0)		13 (54.2)	9 (50.0)	
B	25 (26.3)	6 (33.3)		7 (29.2)	6 (33.3)	
C	11 (11.6)	3 (16.7)		4 (16.7)	3 (16.7)	
Decompensated LC, *n* (%)	26 (27.4%)	5 (27.8%)	0.97	7 (29.2%)	5 (27.8%)	0.92
ALT (IU/L)	170 ± 181	128 ± 82	<0.001	155 ± 168	128 ± 82	0.1
CR (mg/dL)	0.82 ± 0.15	0.79 ± 0.18	0.24	0.79 ± 0.16	0.79 ± 0.18	0.97

*Values are expressed as mean ± standard deviation or numbers (%). LC, liver cirrhosis; ALT, alanine transaminase; CR, creatinine*.

### Virological Response

Over the 24-month follow-up, TDF and ETV showed similar effectiveness in achieving CVR after PSM (6.5 vs. 12.9% at 1 month, 40.3 vs. 46.0% at 3 months, 53.2 vs. 52.4% at 6 months, 64.5 vs. 66.1% at 12 months, 73.4 vs. 72.6% at 18 months, 83.9 vs. 75.0% at 24 months, *p* > 0.05). For HBeAg-positive patients in TDF group (*n* = 108), CVR rate at 1, 3, 6, 12, 18, and 24 months of treatment were 3.7, 37, 50, 59.3, 69.4, and 81.5%, while the corresponding response rates were 6.7, 39, 44.8, 63.8, 71.4, and 74.3% in ETV group (*n* = 105). There was no significant difference in CVR between groups for HBeAg-negative patients at 1, 3, 6, 12, 18, and 24 months [25, 62.5, 75, 100, 100, and 100% in TDF group (*n* = 16), while 47.4, 84.2, 94.7, 78.9, 78.9, and 78.9% in ETV group (*n* = 19)]. Subgroup analysis showed that for patients with high HBV DNA level (>6 log10 IU/mL), the cumulative rate of achieving CVR cat 12 and 24 months were 64.8% (70/108) and 83.3% (90/108) in TDF group, while the corresponding CVR rate were 64.7% (66/102) and 72.5% (74/102) in ETV group.

**Figure 1:**
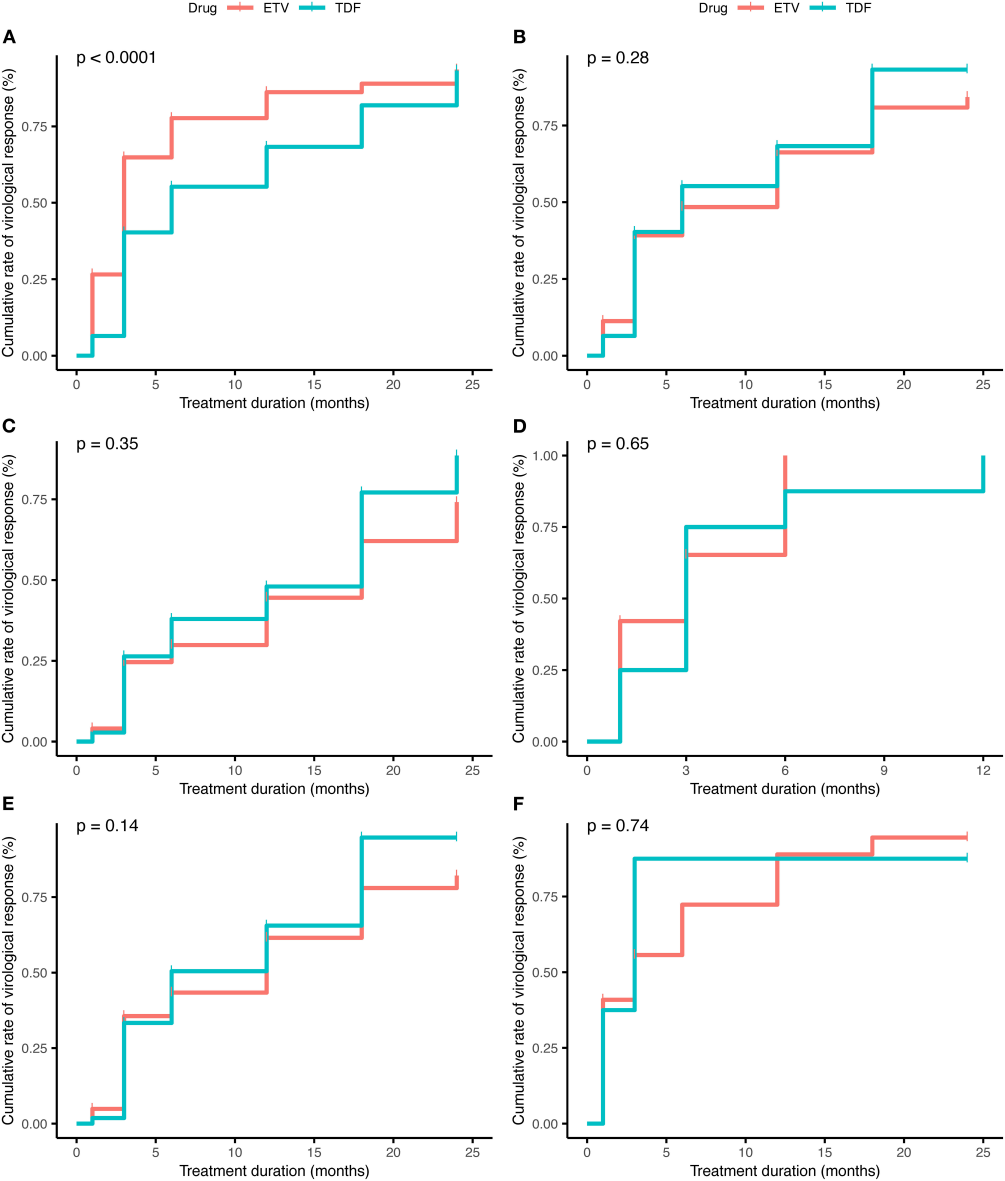
The decline of HBV DNA level of **(A)** 124 patients treated with TDF, **(B)** 124 patients treated with ETV, **(C)** HBeAg-positive patients treated with TDF, **(D)** HBeAg-positive patients treated with ETV, **(E)** patients with high HBV DNA (≥6 log10 IU/mL) treated with TDF, **(F)** patients with high HBV DNA (≥6 log10 IU/mL) treated with ETV.

**Figure 2:**
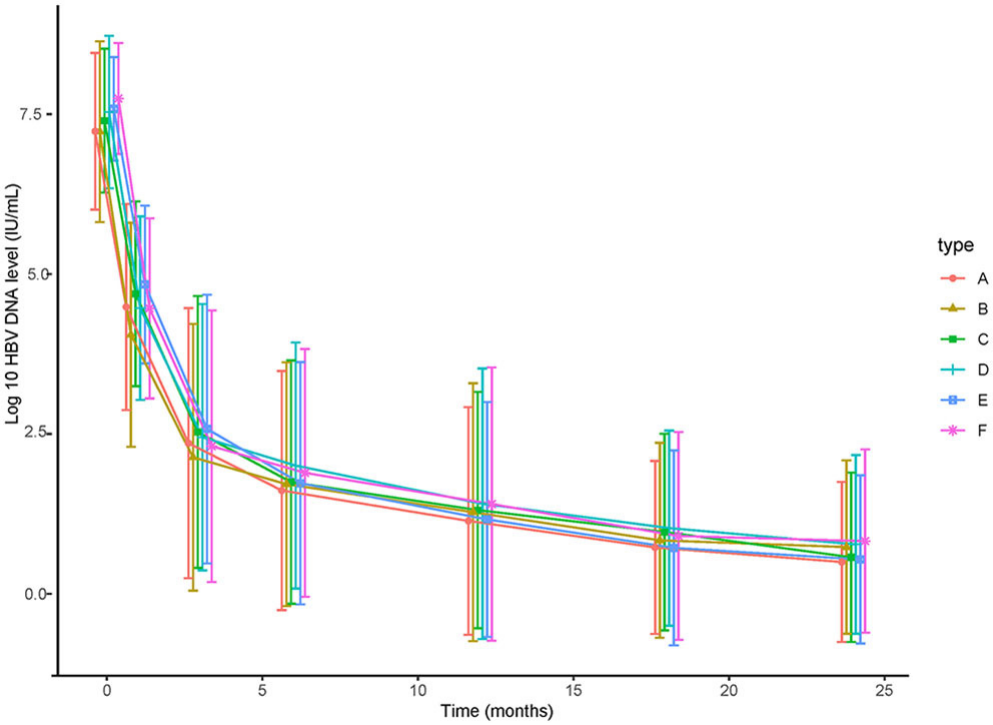
The decline of HBsAg level of (A) 124 patients treated with TDF, (B) 124 patients treated with ETV, (C) HBeAg-positive patients treated with TDF, (D) HBeAg-positive patients treated with ETV, (E) patients with high HBsAg (≥3 log 10 IU/mL) treated with TDF, (F) patients with high HBsAg (≥3 log 10 IU/mL) treated with ETV.

Univariate and multivariate analyses revealed that lower baseline HBV DNA (HR 1.707, 95% CI 1.131–2.577, *p* < 0.01), EVR (HR 3.154, 95% CI 2.268–4.367, *p* < 0.001) and HBeAg-negative status (HR 1.739, 95% CI 1.102–2.744, *p* = 0.02) were independent protective factors of CVR in the cohort study(shown in Table 2). The result of Kaplan–Meier analysis was presented in Figure 1 while the reduction of HBV DNA was shown in Figure 2, all the *p*-values were >0.05.

**Table 2:** Univariate and multivariate analysis for CVR in matched patients.

	**Univariate analysis**			**Multivariate analysis**		
**Predictors**	**HR**	**95% CI**	***p-*value**	**HR**	**95% CI**	***p-*value**
Drug (ETV vs. TDF)	0.881	0.665–1.167	0.38			
Sex (female vs. male)	1.326	0.996–1.767	0.05	1.244	0.932–1.661	0.14
Age (young vs. old)	1.007	0.992–1.022	0.374			
EVR (yes vs. no)	2.066	2.558–4.739	<0.001	1.739	2.268–4.367	<0.001
High HBV level (no vs. yes)	2.023	1.388–2.948	<0.001	1.707	1.131–2.577	0.01
HBeAg (no vs. yes)	2.913	1.947–4.358	<0.001	1.739	1.102–2.744	0.02
Cirrhosis (no vs. yes)	1.475	0.986–2.209	0.06			
ALT (high vs. low)	1.001	1.000–1.002	0.1			
HBsAg (low vs. high)	1.471	1.241–1.742	<0.001	1.030	0.833–1.272	0.79

*EVR, early virological response; High HBV level, HBV DNA ≥ 6 log10 IU/mL*.

### Virological Breakthrough and Drug Resistance

During the course of therapy, 15 patients (12.1%) and 7 patients (13%) in TDF group and ETV group experienced VBT, respectively (*p* = 0.85). Among these patients tested for mutations, 1 patient developed ETV-resistant mutations of rtL180M, rtT184A, and rtM204V, while no patient in the TDF group developed TDF-resistant mutation. After systematic review of electronic records, the high incidence of VBT might be attributed to poor treatment adherence.

### Serological Response

In TDF group (*n* = 108), 1.9, 7.4, 7.4, 13.9, 14.8, 18.5, and 20.4% of patients achieved HBeAg seroclearance at 1, 3, 6, 9, 12, 18, and 24 months of treatment, respectively. Accordingly, 1.0, 2.9, 2.9, 7.6, 10.9, 17.1, and 19.0% in ETV group (*n* = 105) achieved HBeAg seroclearance. The difference in cumulative possibility of HBeAg seroclearance between groups was not significant at each time point. Univariate and multivariate analyses (shown Table 3) revealed that female (HR 1.923, 95% CI 1.029–3.597, *p* < 0.04), younger age (HR 1.078, 95% CI 1.024–1.135, *p* < 0.01), EVR (HR 4.545, 95% CI 2.268–4.367, *p* < 0.001), and lower baseline HBeAg level (HR 1.002, 95% CI 1.001–1.004, *p* < 0.03) were independent protective factors of HBeAg seroclearance in the cohort study (shown in Table 3).

**Table 3:** Univariate and multivariate analysis for HBeAg seroclearence in matched patients.

	**Univariate analysis**			**Multivariate analysis**		
**Predictors**	**HR**	**95% CI**	***p*-value**	**HR**	**95% CI**	***p*-value**
Drug (ETV vs. TDF)	0.861	0.470–1.577	0.627			
Sex (female vs. male)	2.427	1.311–4.505	<0.01	1.923	1.029–3.597	0.04
Age (young vs. old)	1.085	1.026–1.147	<0.01	1.078	1.024–1.135	<0.01
EVR (yes vs. no)	5.814	2.584–13.158	<0.001	4.545	1.984–10.417	<0.001
High HBV level (no vs. yes)	1.627	0.685–3.862	0.27			
HBeAg (low vs. high)	1.002	1.001–1.004	0.01	1.002	1.001–1.004	0.03
Cirrhosis (no vs. yes)	7.931	1.091–57.665	0.06			
ALT (high vs. low)	1.002	1.001–1.003	0.15			

**Figure 3:**
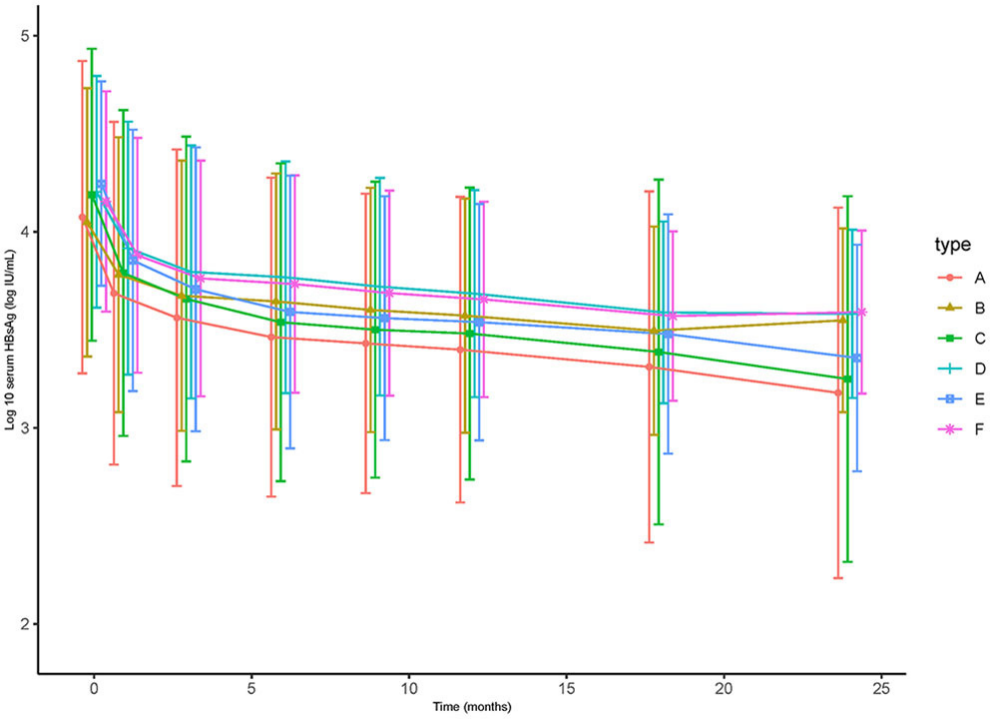
The cumulative rate of complete virological response of (A) the total of 414 patients (*p* > 0.05), (B) matched 124 pairs of patients (*p* > 0.05), (C) HBeAg-positive patients (*p* > 0.05), (D) HBeAg-negative patients (*p* > 0.05), (E) patients with high HBV DNA (≥6 log10 IU/mL) (*p* > 0.05), (F) patients with low HBV DNA (<6 log10 IU/mL) (*p* > 0.05).

As for HBsAg seroclearance, only 2 patients in TDF group achieved HBsAg seroclearance, while none of patient in ETV-treated group. Further analysis showed that more patients in TDF group achieved HBsAg partial serological response than ETV (29.6 vs. 12.4% at 6 months, 33.3 vs. 17.1% at 12 months, 39.8 vs. 24.8% at 18 months, 44.4 vs. 29.5% at 24 months, *p* < 0.05). Figure 3 showed the reduction of HBsAg in matched patients, HBeAg-positive patients, and patients with high baseline HBsAg level (>3 log IU/mL). Notably, mean reduction of HBsAg in TDF group was significantly higher than that in ETV at each time point (0.39 vs. 0.27 log IU/mL at 1 month, 0.51 vs. 0.37 log IU/mL at 3 months, 0.61 vs. 0.40 log IU/mL at 6 months, 0.64 vs. 0.45 log IU/mL at 9 months, 0.68 vs. 0.48 log IU/mL at 12 months, 0.76 vs. 0.55 log IU/mL at 18 months, 0.91 vs. 0.66 log IU/mL at 24 months, *p* < 0.05). In subgroup analysis, we found that the significant difference still existed in patients with high baseline HBsAg level, indicating that TDF might be more effective in decreasing HBsAg in patients with higher HBsAg level. Mean reduction at 1, 3, 6, 9, 12, 18, 24 months was 0.39, 0.54, 0.65, 0.69, 0.71, 077, and 0.89 log IU/mL in TDF-treated patients with high baseline HBsAg level, respectively; the corresponding reduction in ETV-treated patients was 0.27, 0.39, 0.42, 0.47, 0.50, 0.58, and 0.56 log IU/mL, respectively (*p* < 0.05). Univariate and multivariate analyses (shown in Table 4) revealed that patients treated with TDF (HR 1.724, 95% CI 1.134–2.625, *p* < 0.01) without LC at baseline (HR 2.182, 95% CI 1.044–4.557, *p* < 0.04), with lower baseline HBsAg level (HR 2.169, 95% CI 1.427–3.297, *p* < 0.001) were likely to achieve HBsAg seroclearance.

**Table 4:** Univariate and multivariate analysis for partial HBsAg seroclearence in matched patients.

	**Univariate analysis**			**Multivariate analysis**		
**Predictors**	**HR**	**95% CI**	***p*-value**	**HR**	**95% CI**	***p-*value**
Drug (TDF vs. ETV)	1.754	1.156–2.660	<0.01	1.724	1.134–2.625	0.01
Sex (female vs. male)	1.075	0.710–1.629	0.73			
Age (young vs. old)	0.977	0.953–1.002	0.07			
EVR (yes vs.no)	1.114	0.740–1.675	0.607			
High HBV level (no vs. yes)	0.367	0.160–0.839	0.02	0.945	0.382–2.340	0.9
HBeAg (no vs. yes)	0.252	0.093–0.687	<0.01	0.542	0.178–1.654	0.28
Cirrhosis (no vs. yes)	2.476	1.199–5.112	0.01	2.182	1.044–4.557	0.04
ALT (high vs. low)	1.001	1.000–1.002	0.08			
HBsAg (low vs. high)	2.489	1.699–3.647	<0.001	2.169	1.427–3.297	<0.001

### Biological Response and Safety

The proportion of patients achieving BR was compared between TDF and ETV group, with no significant difference observed during the course of therapy. At 3, 6, 12, and 24 months, normalization of ALT was achieved in 51, 68.6, 81.8, and 83.8% patients treated with TDF and in 62.5, 79.6, 81.1, and 89.4% patients treated with ETV (*p* > 0.05), respectively. The difference in ALT normalization between groups was not significant in HBeAg-positive or HBeAg negative patients. There were 4 patients in the TDF group and 2 patients in the ETV group experienced ALT flare, of whom 1 patient administrated with ETV experienced drug resistance.

We recorded baseline and maximal value of SCr in the 24-month follow-up period. There was no patient experienced AKI, with mean increase of SCr was 0.10 and 0.08 mg/dL in TDF and ETV group (*p* = 0.11), respectively. Additionally, 11 (8.8%) and 8 (6.5%) of patients in the TDF and ETV group experienced transient hypophosphatemia during the treatment, but none had subjective symptoms, such as poor appetite and musculoskeletal pain. No patient was observed to cease or change antiviral therapy due to side effects during the whole treatment period.

### LC and HCC Development

During the 2-year follow-up, periodically monitored imaging examinations and alpha-fetoprotein diagnosed 2 ETV-treated patients with HCC after more than 6-month drug treatment, one of whom had LC at baseline. Two patients were older than 40 and achieved BR after 3 months. Both patients had high HBV DNA level (8.21 and 8.97 log10 IU/mL), and achieved CVR after 18-month therapy, indicating poor effect of ETV in these 2 patients. Furthermore, both patients had high baseline HBsAg, and neither of them achieved HBeAg or HBsAg seroclearance. In addition, 2 patients in ETV group were detected to develop LC during the 2-year follow-up, with no patient in TDF group. Despite these 2 patients were HBeAg-positive and had high HBV DNA (8.29 and 8.24 log10 IU/mL) and HBsAg level (3.49 and 5.10 log IU/mL) at treatment initiation, both of them achieved sustained virological response and sustained BR after 3-month antiviral therapy, but neither of them achieved HBeAg or HBsAg seroclearance. Univariate and multivariate analyses revealed that there was no independent factor in the development of HCC and LC.

## Discussion

NAs for CHB have been evolved over two decades, with agents becoming more effective and safer, of which ETV and TDF are equally recommended as the first-line oral antiviral currently. Long-term therapeutic effects of ETV and TDF in suppressing HBV replication, reducing liver necroinflammation and incidence of HCC have been verified by studies (31–33). However, there are still some controversies about the effectiveness between these two drugs. Therefore, we comprehensively compared long-term virological response, change of HBV DNA, BR, serological response, incidence of HCC and LC, and side effect over time between drugs, especially dynamic change of HBsAg in the curse of treatment.

We are the first to conduct a period of 2 years of comparison research on HBsAg complete and partial serological response, and dynamic reduction. As the outer shell protein of HBV, HBsAg does not have infectious, but its appearance often accompanies the existence of HBV (34). Therefore, HBsAg is the sign indicating infection with HBV. Functional cure is defined as the seroclearance of HBsAg and the presence of anti-HBsAg, which is the current goal of antiviral therapy. Functional cure occurs less frequently in patients treated with NAs when compared with interferon, although NA is more effective at inhibiting HBV DNA (35). Interestingly, we found cumulative rate of partial serological response was significantly higher in TDF group at each time point, and similarly, HBsAg reduction in the TDF group was also more significant higher at 1, 3, 6, 9, 12, 18, 24 months. Besides, TDF was the positive independent factor of HBsAg partial seroclearance according to univariate and multivariate analyses. We hold that TDF is more effective in reducing HBsAg level, despite the comparable seroclearance in two drugs. Similar result was also found in former published study, revealing that HBsAg reduction was higher in HBeAg-negative CHB patients switching to TDF than continuing ETV therapy (30). Furthermore, a *vitro* experiment reported an additional pharmacological effect of nucleotide analogs (adefovir or TFD) by inducing IFN-λ3 production, further inducing IFN-stimulated genes and resulting in a reduction of HBsAg production (36). It is acknowledged that HBsAg seroclearance further reduces hepatocellular carcinoma risk after complete viral suppression with NAs (37). In addition, large-scale cohort study and meta-analyses showed that TDF was more likely to reduce the risk of developing HCC especially in LC patients (15, 27–29). Therefore, we can make reasonable conjecture that TDF can lower the risk of HCC by achieving larger reduction of HBsAg, which might be illustrated by higher level of IFN-λ3 in TDF-administrated patients.

Although development of HCC in our study was not significantly associated with drug treatment or other factors, two reasons might account for it. On the one hand, old age and LC were seen as risk factors of HCC, but there were a few of LC patients at baseline, and the baseline age in our study was slightly young after propensity-score matching (15, 38). On the other hand, 2-year follow-up time might not be long enough for progression in HCC. The effect in reducing HBsAg and association between HBsAg and HCC necessitate additional validation in a more extensive and multiple-center cohort, and more mechanistic investigation were needed.

In this prospective cohort study, TDF was not superior to ETV in achieving complete viral suppression at any inspection time during the 2-year treatment, which was in accordance with previous studies (39, 40). Same result was seen in HBeAg-positive or high- viremia patients. It is worth noting that the rate of VBT in our cohort study was quite high, mostly due to poor patient compliance. Experiencing virological failure with drug resistance is a prognostic sign for poorer long-term clinical outcome. Therefore, patient education and practice should be urgently strengthened for CHB patients, especially for less educated patients, in order to improved long-term prognosis.

As for the adverse effect, some previous studies reported that TDF could have nephrotoxicity during the treatment (41, 42). However, TDF showed potent security in our study, with no patient experienced AKI in both groups and comparable increase of SCr with ETV group. However, 8.8 and 6.5% of patients in the TDF and ETV group experienced transient hypophosphatemia during the treatment, but none of them had subjective symptoms, such as poor appetite and musculoskeletal pain. It might be attributed to diet, electrolyte or renal function, etc. Although hypophosphatemia might have no critical influence on treatment, more attention and thinking should be paid for the emergence of hypophosphatemia.

There are a few limitations to this study. First, this is not a long-term but an intermediate-term study, and all the conclusions were drawn based on the limited 2-year observation. A long-term study would be able to draw more definitive conclusions. Moreover, this is a single-center, observational study, so similar studies should be replicated in multicenter and prospective studies to validate and generalize our study's findings. Thirdly, although we used PSM to minimize selection bias, matched patients were characterized as younger age and low rate of baseline LC. Therefore, progression in liver disease was seldom detected in our study. In addition, retrospective study is a prospective study that began at some point in the past. Therefore, not all details of patients were recorded, or could be easily to be accessed, such as information of taking medication, which might result in the slightly high rate of virological breakthrough.

Although CVR, BR, HBeAg seroclearance, and incidence of hepatocellular carcinoma are equivalent with TDF and ETV, TDF has higher potency in reducing HBsAg than ETV. Considering the effect still existed in patients with high HBsAg level (>3 log IU/mL) and HBeAg seropositivity, TDF might be a superior therapeutic regimen combining with its relatively safety.

## Data Availability Statement

The raw data supporting the conclusions of this article will be made available by the authors, without undue reservation.

## Ethics Statement

The studies involving human participants were reviewed and approved by The Ethics Committee of the First Affiliated Hospital of College of Medicine of Zhejiang University (IIT20200407A). Written informed consent for participation was not required for this study in accordance with the national legislation and the institutional requirements.

## Author Contributions

SY and CY designed the research. SY, XM, and CC performed the study. SY and HW analyzed the data and wrote the manuscript. FX, XM, and CC revised the manuscript. CY provided technical support. All authors contributed to the article and approved the submitted version.

## Conflict of Interest

The authors declare that the research was conducted in the absence of any commercial or financial relationships that could be construed as a potential conflict of interest.

## Abbreviations

HBV: hepatitis B virus
CHB: chronic hepatitis B
HCC: hepatocellular carcinoma
HBeAg: hepatitis B e antigen
HBsAg: hepatitis B surface antigen
cccDNA: covalently closed circular DNA
ETV: entecavir
TDF: tenofovir disoproxil fumarate
NA: nucleos(t)ide analog
CVR: complete virological response.
